# Keeping the Beat: A Large Sample Study of Bouncing and Clapping to Music

**DOI:** 10.1371/journal.pone.0160178

**Published:** 2016-07-29

**Authors:** Pauline Tranchant, Dominique T. Vuvan, Isabelle Peretz

**Affiliations:** 1 Département de psychologie, Université de Montréal, Montréal, Québec, Canada; 2 International Laboratory for Brain, Music and Sound Research (BRAMS), Université de Montréal, Montréal, Québec, Canada; University of California Merced, UNITED STATES

## Abstract

The vast majority of humans move in time with a musical beat. This behaviour has been mostly studied through finger-tapping synchronization. Here, we evaluate naturalistic synchronization responses to music–bouncing and clapping–in 100 university students. Their ability to match the period of their bounces and claps to those of a metronome and musical clips varying in beat saliency was assessed. In general, clapping was better synchronized with the beat than bouncing, suggesting that the choice of a specific movement type is an important factor to consider in the study of sensorimotor synchronization processes. Performance improved as a function of beat saliency, indicating that beat abstraction plays a significant role in synchronization. Fourteen percent of the population exhibited marked difficulties with matching the beat. Yet, at a group level, poor synchronizers showed similar sensitivity to movement type and beat saliency as normal synchronizers. These results suggest the presence of quantitative rather than qualitative variations when losing the beat.

## Introduction

Humans move to musical rhythms by nodding the head, clapping the hands or dancing in time with perceived periodicities in musical stimuli–that is, with the musical beat. Such movements are spontaneous and observed across cultures [1]. Infants show a rhythmic motor response to music before the age of two [2] but it is only between the ages of 2.5 and 4.5 that the flexibility required to match their movement tempo to those of the stimuli starts to develop [3].The behavioural and neural mechanisms required by the capacity for rhythmic sensorimotor synchronization (SMS) are presently the topic of intense research (see [4,5] for reviews).

The vast majority of the studies conducted in this area have investigated finger tapping. Yet, finger tapping leaves out important aspects of the processes involved in SMS to music, such as the diversity of natural movements and the feedback they provide, as well as the pleasurable aspect of moving to music.

Different movements may recruit different mechanisms. For example, different timing and motor control mechanisms may underlie the production of continuous versus discrete periodic movements (such as tapping). Emergent properties of the movement dynamics and the representation of event-based timing seem to differentiate continuous from discrete movements [6–9]. This distinction is supported by several findings. Temporal precision in finger tapping and continuous drawing is not related within individuals [10,11], and different autocorrelation patterns characterize the period and asynchronies for discrete versus continuous synchronization [12,13]. Furthermore, patients with cerebellar damage show preserved circle drawing (continuous) but impaired finger tapping (discrete) when asked to time their movement to isochronous tones [14].

Second, vestibular stimulation, which varies when bending the knees to move the trunk up and down in bouncing, is constant in finger tapping and clapping. Yet vestibular stimulation appears to drive beat finding in music. Indeed, bouncing to a specific meter while listening to a rhythmic pattern can affect perceptual meter judgments [15,16]. This is also found for motion of the head only (as opposed to the legs) and whole-body passive motion [17], suggesting that it is the manipulation of vestibular information that is playing a crucial role.

Last but not least, the desire to move to certain types of music, a phenomenon referred to as groove [18], is a highly pleasurable experience [19]. When exposed to music, people spontaneously start to move their foot, head and/or trunk [20]. Preventing listeners from moving their body actually reduces their ability to find the beat [21]. Moreover, participants prefer to move freely with music rather than to be directed to make hand-tapping movements only [20]. Restricting a participant’s movements such that they may only move their finger, as is the case in tapping studies, is likely to restrain this participant’s feeling of 'being in the groove'. This is not only because participants are required by the experiment to perform a specific gesture, but also because that gesture may not be one that arises spontaneously in non-experimental contexts. As groove is an important aspect of motor engagement during music listening [22] and of the quality of sensorimotor coupling [20], the experimental study of movements that occur spontaneously outside of the lab may help improve the understanding of some important aspects of SMS to music in humans.

In sum, the way we move has an influence on how we interpret, enjoy and synchronize to musical rhythms. A few studies have explored the effects of using different effectors, such as finger versus foot [23] or finger versus drumstick [24] on the quality of isochronous synchronization, and there is a growing literature on the synchronization of gait (e.g. [25,26]) and dance-like movements (e.g. [16,27,28]) to music. However, no studies so far have compared different forms of naturalistic but qualitatively distinct movement, such as bouncing and clapping during synchronization to music. This comparison was the primary goal of the current study.

A second goal of the current study was to explore the effect of beat saliency on clapping and bouncing. Synchronization to music requires the perceptual encoding of a periodic beat structure from the musical stimulus. This beat structure is neither consistently periodic in reality [29] nor does it systematically contain acoustic energy at beat locations, such as in syncopated rhythms [30,31]. Since there is no one-to-one relationship between beats and sounded events in music, an important task for the perceptual system is to track the beat in music in order to synchronize to it. Moreover, there are considerable variations in beat saliency (i.e., the perceptual clarity of the beat) across musical genres. Compare, for example, techno dance in which there is generally a strong bass kick marking each beat, to jazz, in which the beat is much more subtle.

Previous studies have assessed the effect of rhythmic complexity, which is conceptually related to beat saliency, on synchronization by comparing metrical to non-metrical rhythmic sequences [32,33] and by comparing metronome to musical sequences [34,35]. These studies generally show better synchronization for lower rhythmic complexity. In a similar vein, Fitch and Rosenfeld [30] studied how syncopation in single-tone rhythms, i.e., “rhythmic events that violate one’s metrical expectations” [19] affected synchronization, and showed that higher tapping accuracy was associated with lower degrees of syncopation. Specifically, increasing the number of un-syncopated isochronous “streams” in computer-generated rhythms tends to improve tapping accuracy [36].

Less is known about the role of beat saliency on synchronization. Chen et al. [32] and more recently Fujii and Schlaug [37] assessed the effect of beat saliency on tapping behaviour by periodically increasing the intensity of isochronous tones to create accent patterns. In both studies, the louder (more salient) accents led participants to increase the duration [32] and pressure [37] of their taps. In the same vein, Burger et al. [38] found that beat saliency ("pulse clarity" in their paper) positively correlates with the amount and speed of movement. However, in these three studies the accuracy of synchronization was not assessed. Van Dyck et al [28] showed that better tempo entrainment of the head is found when increasing the sound level of the bass drum in a club-like dance context. Finally, better period-matching with a beat is found for high- compared to low-groove music [20,25]. Although beat saliency has been associated with groove in music [20,39], these two concepts may recruit beat finding mechanisms differently. Indeed, syncopated rhythms have been shown to elicit a higher perception of groove than rhythms with a straight-ahead beat [19]. For example, a metronome has the most salient beat but is not groovy. Moreover, groove is bound to movement whereas beat saliency relates to perception. Therefore, in the current study we used stimuli that varied in beat saliency in order to gain insights into the perceptual component of SMS. Keeping in line with our attempt to study SMS in a naturalistic context, our goal was to evaluate synchronization to naturalistic musical stimuli (i.e., commercially available music) that were distributed along a spectrum of beat saliency.

To these aims, we tested 100 healthy young adults on their ability to match the tempo of musical pieces when asked to bounce or clap the hands in time with the beat of the stimuli. We predicted the occurrence of synchronization difficulties in a minority of individuals, as previously found with finger-tapping [35] and bouncing [34]. Rather than excluding the data of such individuals, as is usually the case in SMS studies, we applied a neuropsychological approach to study these impaired cases as a way of gaining insight into the mechanisms of normal synchronization.

## Materials and Methods

### Experiment

A large group of young unselected university students were invited to bounce and clap to a set of music excerpts varying in beat saliency. We also measured their ability to (a) maintain a regular movement without music and (b) to perceptually infer the meter from short musical excerpts taken from the Montreal Battery of Evaluation of Amusia (the metric test; [40]).

### Participants

We tested 101 healthy university students (Aged 18–34, M = 23.4; 56 female) who provided written informed consent and received financial compensation for their participation. None of them reported any neurological problems or motor deficits. They all self-reported having normal hearing. A description of their musical and dance background is presented in Table 1.

**Table 1:** Musical and dance experience of participants.

Musical training	Number of participants
No training	29
Self-learned (more than 7 years of practice)	7
0.5 to 5 years of formal music classes	39
More than 5 years of formal music classes	20
Professional musicians and graduate music students	6
**Dance training**	
No training	62
0.5 to 5 years of formal dance classes	25
More than 5 years of formal dance classes	13

All participants completed the on-line test of amusia [41] to screen for music perceptual difficulties, and completed the MBEA metric test in the lab [40]. The on-line test assesses out-of-key tone discrimination and off-beat detection, both in a melodic context. In the MBEA metric test, participants are asked to find the underlying pattern of strong and weak beats in 32 piano sequences, in order to judge them as being marches (strong beat on every other beat) or waltzes (strong beat on every third beat). Nine individuals obtained poor scores on the on-line pitch tests and were further tested with the entire MBEA. One participant obtained a melodic composite score (i.e. mean of the scale, contour, and interval tests, each comprising 30 trials) of less than 22 (out of 30). This participant was hence diagnosed with “pitch-deafness” and was therefore excluded from the study. Thus, the final sample included 100 individuals with no perceptual pitch impairment. The research protocol was approved by the Comité d’éthique de la recherche de la Faculté des arts et des sciences (CÉRFAS) at Université de Montréal.

### Stimuli

There were six musical stimuli varying in musical style and tempo and two metronomes. Detailed descriptions of the stimuli are presented in Table 2. We selected four stimuli previously used to measure bouncing synchronization in 33 good and one poor synchronizers [34]. These were Metronome (116 and 125 BPM), Suavemente (Merengue, 116 and 124 BPM), What a Feeling (Pop dance) and The Flow (Dance lounge). Merengue and Metronome stimuli were presented at two different tempi in order to assess the effect of tempo on synchronization. Stimuli were constructed by looping initial selections at a stable tempo, so that the period between the last beat of any one excerpt and the first beat of the next excerpt in the loop was equal to the inter-beat period, maintaining the beat across excerpts for 115 seconds. In order to vary musical genres as well as beat saliency, we added two stimuli that also had previously been used in our lab (unpublished data): Since You've Been Gone (Soul) and Brand New Carpet (Pop rock). All stimuli were chosen in a relatively narrow tempo range, typical of dance music [42] and around people's perceptual preferred tempo (120 BPM, see [43]). The inter-beat intervals (IBIs) varied from 455 to 517 milliseconds, corresponding to tempi between 116 and 132 BPM. The tempi were determined using the mirtempo function from the MIR toolbox [44] in Matlab (MathWorks).

**Table 2:** Characteristics of stimuli.

Stimulus name	Genre	Tempo (BPM)	Familiarity (Z-score)	Beat Saliency (Z-score)
Metronome	-	125/116		1.14
What a feeling^**1**^	Pop dance	132	-0.50	0.49
The flow^**1**^	Dance lounge	120	-0.40	0.41
Suavamente^**1**^	Merengue	124/116	1.16	-0.13
Brand new carpet	Pop rock	126	-0.68	-0.76
Since you've been gone	Soul	117	0.42	-1.15

^**1**^ musical excerpts derived from [34].

In order to best map the relation between beat saliency and synchronization performance, we decided to derive an empirical measure of beat saliency from participants with similar levels of musical training as those in the synchronization experiment. This method was favoured over acoustical analysis methods that have been developed and validated using trained musicians' ratings (for example the mirpulseclarity function in the MIR toolbox). To do so, 14 university students (Aged 20–26, *M* = 22.3, 7 female) who did not take part in the bouncing and clapping synchronization experiment but were screened according to the same criteria, participated in an additional pilot study. They were asked to indicate ‘how clear and salient’ the beat of each stimulus was by moving a slider potentiometer (10 K Ohm, 0.5W, 10 mm) controlled by customized Python scripts from left to right while listening to the stimulus. The slider value ranged from zero (least salient beat) to 1023 (most salient beat). Z-scores for each stimulus are provided in Table 2. Paired-sample t-tests revealed no difference between the two Merengue stimuli (*t*(13) = -0.4, *p* = 0.66) or between the two Metronome stimuli (*t*(13) = 0.6, *p* = 0.58), indicating that the tempo difference did not affect the saliency of the perceived beat. Therefore, the values were collapsed over the two tempi, producing a single value for the Merengue and Metronome (Table 2). A one-way repeated-measures ANOVA with 6 levels (Merengue, Metronome, Dance lounge, Pop Dance, Soul and Pop Rock) for the Stimulus factor revealed a significant effect of Stimulus, *F*(5,65) = 28.2, *p* < 0.001. There was a significant linear trend in the Stimulus factor, *t*(78) = 10.1, *p* < 0.0001.

Finally, familiarity with the musical stimuli was assessed in a separate pilot study including 27 participants (aged 20–38, M = 25.6, 18 female) who did not take part in the synchronization experiment or the beat saliency experiment but were screened according to the same criteria. They rated how familiar they were with the stimuli on a 1 to 100 scale (1 = not familiar at all, 100 = very familiar). Responses were made on the keyboard. Results revealed that two of the stimuli, Suavemente (Merengue) and Since You've Been Gone (Soul), were more familiar than the others (Table 2).

### Bouncing and clapping synchronization

Movement condition presentation order was counter-balanced between participants, with half of the participants starting with the bouncing and the other half starting with the clapping. Each participant received the same set of stimuli. We kept the order of stimulus presentation constant across tasks and participants (except for the two Merengue and Metronome that were counter-balanced for tempo). The stimuli presentation order was as follows: Merengue (two tempi), Metronome (two tempi), Pop Dance, Dance Lounge, Soul, Pop Rock. The main reason for this design was that the musical selections vary in beat saliency, and this variation may have unpredictable carry-over effects. For example, a musical stimulus with an easy beat to track may prime beat finding in the next excerpt; conversely, a difficult beat to track in one musical stimulus may impair beat tracking in the next excerpt. Since our main goal was to compare bouncing and clapping, we used the same order of presentation for the stimuli in both tasks. This procedure insured that any carry-over effects that existed between stimuli would be stable across the tasks, and any carry-over effects that existed between tasks would cancel out across participants. Participants were instructed to move in time with the ‘strong and regular beat’ of the stimulus and to continue the same movement throughout the stimulus. Before starting each synchronization condition they were asked to bounce or clap “regularly at their own preferred rate”, for two minutes. The testing session lasted approximately one hour.

### Equipment

The experiment took place in a large sound-attenuated studio, with the experimenter present but facing away from the subject to avoid distraction. Stimuli were presented in free field from Genelec speakers at a comfortable volume level. An accelerometer was used to capture movement. This accelerometer was contained in the remote of a Nintendo Wii and was strapped to the trunk of the subject’s body in the bouncing condition, and to the forearm of the dominant hand in the clapping condition (see S1 Fig). This device continuously measured the acceleration produced by participants’ movements in the three spatial dimensions, at 100 frames per second. Acceleration data were transmitted to an Apple computer via Bluetooth and recorded by a customized program written in MAX (Cycling ‘74). Because of a technical error with the triggering between stimuli and movement capture, phase values provided by our system were not accurate enough for analysis. Nevertheless, we collected data in 1600 files (8 stimuli x 2 movement types x 100 participants). Of these, 7 files were corrupted and were not considered in the analyses.

### Data Analysis and Results

The current section is divided into four parts. In the first part, we report the metrical levels observed in produced movement. In the second part, we describe the procedure we applied to each *trial* (i.e., a participant’s synchronization response to each stimulus) to determine whether participants successfully matched their movements to the tempo (‘Normal Synchronization’) or not (‘Poor Synchronization’). Next, we describe the procedure we applied to each *participant* to determine whether they were a Normal Synchronizer or a Poor Synchronizer. Finally, we analyze synchronization performance separately in normal and poor synchronizers.

### Metrical levels observed in produced movement

Participants produced movement either at the beat level or at the two-beat level. Bounces or claps that occurred at a rate corresponding to the beat frequency were considered to occur at the beat level, whereas bounces or claps that occurred at half the beat frequency (i.e., every second beat) were considered to occur at the two-beat level. For each participant and each stimulus, the level at which the participant moved was determined by calculating the Fourier transform of the acceleration data. Fourier analysis was performed with Matlab. This procedure produced a power spectrum with the maximal peak appearing at the frequency of the participant’s movement.

Two participants produced movement at a very slow rate, close to the four-beat level (beat frequency/4). Further analysis revealed that one of these participants (participant 21) was accurately matching the tempo of all stimuli at the four-beat level: we decided to exclude her in subsequent analyses because her behaviour was not comparable to the rest of the group. In contrast, Participant 18’s responses were not tempo-matched to any of the stimuli. This participant was identified as a ‘Poor Synchronizer’ and excluded from the current analysis. One additional participant produced movement at tempi that did not match the stimulus tempi but from the experimenter's report this was due to an obvious lack of motivation in performing the tasks rather than a synchronization difficulty, and he was excluded from subsequent analysis.

For the remaining 97 participants, the chosen metrical level was not always the same across stimuli. Furthermore, for some trials, it was impossible to determine which level was produced by the participant, because the chosen level was not constant throughout the trial (3 cases) or was produced at a tempo that was not constant or that did not correspond to the beat or two-beat level (48 cases). These trials were excluded. Note that more trials had to be excluded in the bouncing (total of 37 over 20 participants, out of 97*8 = 776 trials) as compared to the clapping condition (total of 14 over 12 participants, out of 776 trials).

The distribution of metrical level (beat-level, two-beat level) displayed in participants’ movements are presented in Table 3. There was a higher proportion of movement at the beat-level (versus two-beat level) in all conditions, and particularly so in the clapping condition (*Mdn* = 82) as compared to the bouncing condition (*Mdn* = 54), *U* = 62.50, *p* < .001). The number of trials on which participants synchronized at the beat versus two-beat level was not affected by the differential effects of the two movement types over the course of the stimulus presentation order, χ^2^(7) = 2.59, *p* = 0.92. Thus, having moved for some time did not impact participants’ chosen synchronization level in a different way for the bouncing compared to the clapping movement.

**Table 3:** Produced metrical levels.

Stimulus, tempo (BPM)	Bouncing		Clapping	
	Beat level^1^	Two-beat level^1^	Beat level^1^	Two-beat level^1^
Metronome, 125	55	39	95	2
Metronome, 116	57	38	89	7
Pop Dance, 132	68	25	85	11
Dance lounge, 120	61	27	90	4
Merengue, 125	52	38	78	16
Merengue, 116	52	38	75	19
Pop, 125	49	44	65	28
Soul, 117	44	47	56	38
**Median**	**54**	**38**	**82**	**14**

^**1**^ Number of participants

### Normal versus poor synchronization

Synchronization performance was quantified by analyzing the acceleration data to determine the degree to which the tempo of the produced movement matched the stimulus tempo. For each stimulus, the first 10 seconds were discarded from the analysis, leaving 105 seconds of data for analysis. First, the timing of maximal flexion of the knee (bouncing) or hand impact (clapping) were extracted from the acceleration data. These time points were called responses and the interval between two responses was called the inter-response interval (IRI).

The response series was analyzed using circular statistics [45,46] and the CircStat Toolbox [47] for Matlab. The timing of the responses was converted to vectors on the unit circle. For beat level synchronization trials, one stimulus inter-beat-interval (IBI) corresponded to one circle length (i.e., 2*pi radians). For two-beat level synchronization trials, two IBIs corresponded to one circle length. For beat level synchronization we segmented each trial into four consecutive segments, while two-beat level synchronization were segmented into two consecutive segments, so that each segment had the same number of responses within a trial. The assessment of synchronization across segments allowed us to catch trials in which the beat was lost after a certain duration, or in which participants required a very long duration to achieve synchronization.

**Fig 1:**
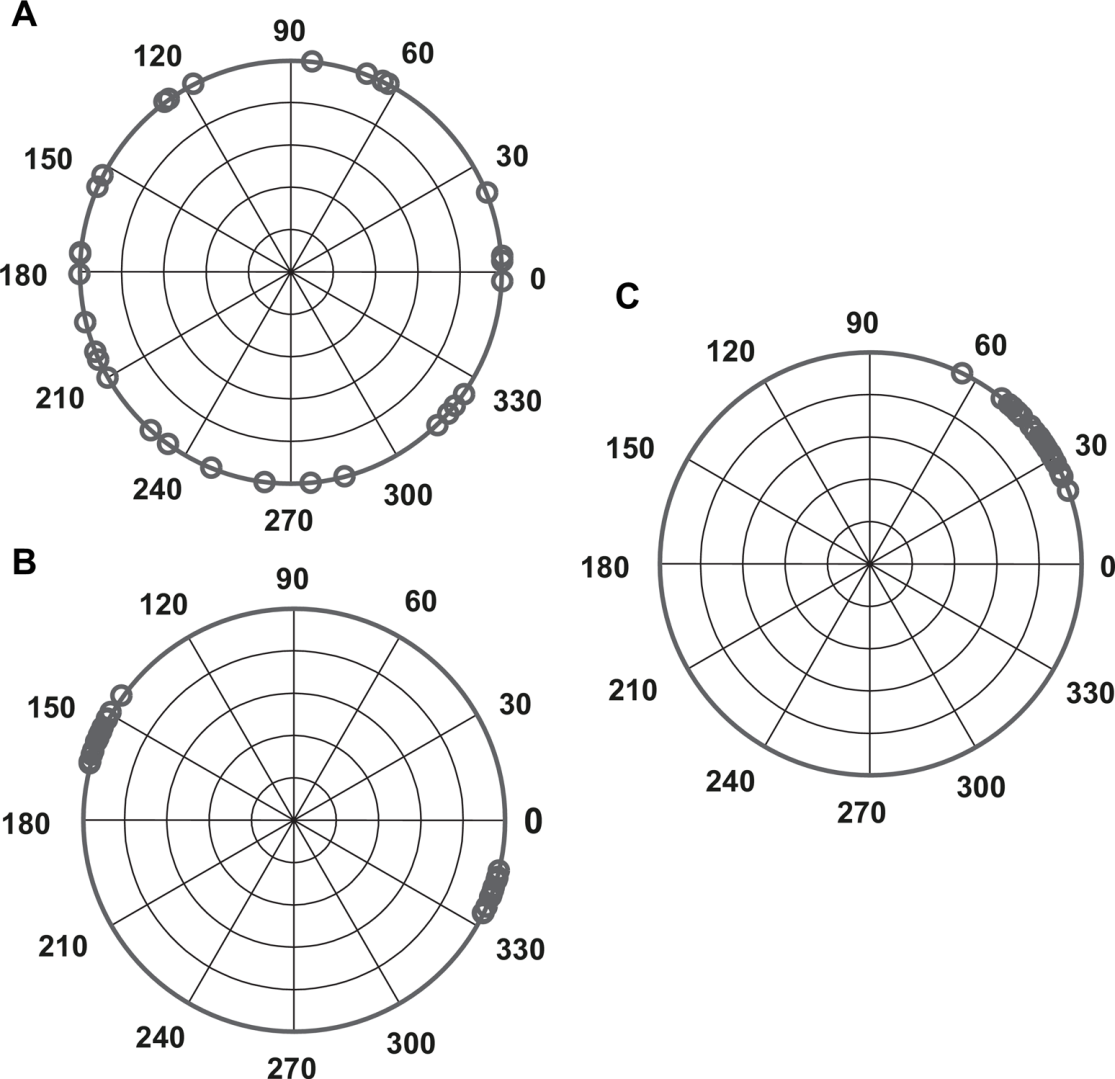
Example distributions of response vectors. A: random distribution, B: bimodal distribution, C: unimodal distribution.

When IRIs are close to the stimulus IBI (or 2*IBI in two-beat synchronization), corresponding vectors are clustered around a preferred direction (Fig 1C). Conversely, if IRIs are consistently different from the stimulus IBI, corresponding vectors are increasingly distributed around the circle (Fig 1A). A Rayleigh test (null hypothesis = random distribution of vectors around the circle) is used to assess whether performance on a particular segment is period-matched to the stimulus. When performance is period-matched (i.e., vectors clustered around a preferred direction, Fig 1C), the Rayleigh test yields a significant result. When performance is not period-matched (i.e., vectors randomly distributed around the circle, Fig 1A), the Rayleigh test yields a non-significant result. Note that the time between responses and beat locations (i.e., phase) was not taken into account here. This means that the preferred direction of the vector series can be located anywhere on the circle. However, in a few trials (20 bouncing and 15 clapping trials) two preferred directions (bimodal distribution) were observed within the trial (Fig 1B). Bi-modal trials were excluded from further analysis. Two more participants were excluded because at least 3 of the 8 clapping or bouncing trials were bimodal, resulting in too much missing data. Finally, each trial for a given participant was categorized as ‘poor synchronization’ if the Rayleigh test was not significant for any one of the segments, and ‘normal synchronization’ if the Rayleigh test was significant for all segments. This procedure detected 53 poor synchronization trials in the bouncing condition (7.4% of trials), and 23 in the clapping condition (3.1% of trials).

A participant was categorized as a ‘Poor Synchronizer’ if s/he had at least 3 poor synchronization trials out of the 6 music trials for at least one movement type (bouncing or clapping). Following this procedure, 13 participants were identified as Poor Synchronizers, in addition to participant 18 (see previous section), yielding 14 Poor Synchronizers. The remaining 82 participants made up the Normal Synchronizers group.

### Normal synchronizers

We analyzed the synchronization data of the 82 normal synchronizers in order to establish a normal profile of performance to which we could compare the performance of poor synchronizers. As in the previous analysis, synchronization failure trials were excluded (18 bouncing and 6 clapping trials). The circular variance, a measure of angular dispersion (see [46] for a description) was calculated for the responses of the entire un-segmented trial, and was used as our index of movement regularity. The distribution of the circular variance, for all stimuli and both movement types, corresponded to a lognormal function. To normalize the distributions, the inverse logarithm of the circular variance was taken as a measure of regularity, with a higher value representing more regularity. This variable will henceforth be referred to as ‘synchronization regularity’ (SR). A description of log-transformed SR scores are provided in S1 Table. There was no correlation between SR (all stimuli averaged) and musical training or dance training, and as those factors were not of primary interest in the present study they were not included in our statistical model.

**Fig 2:**
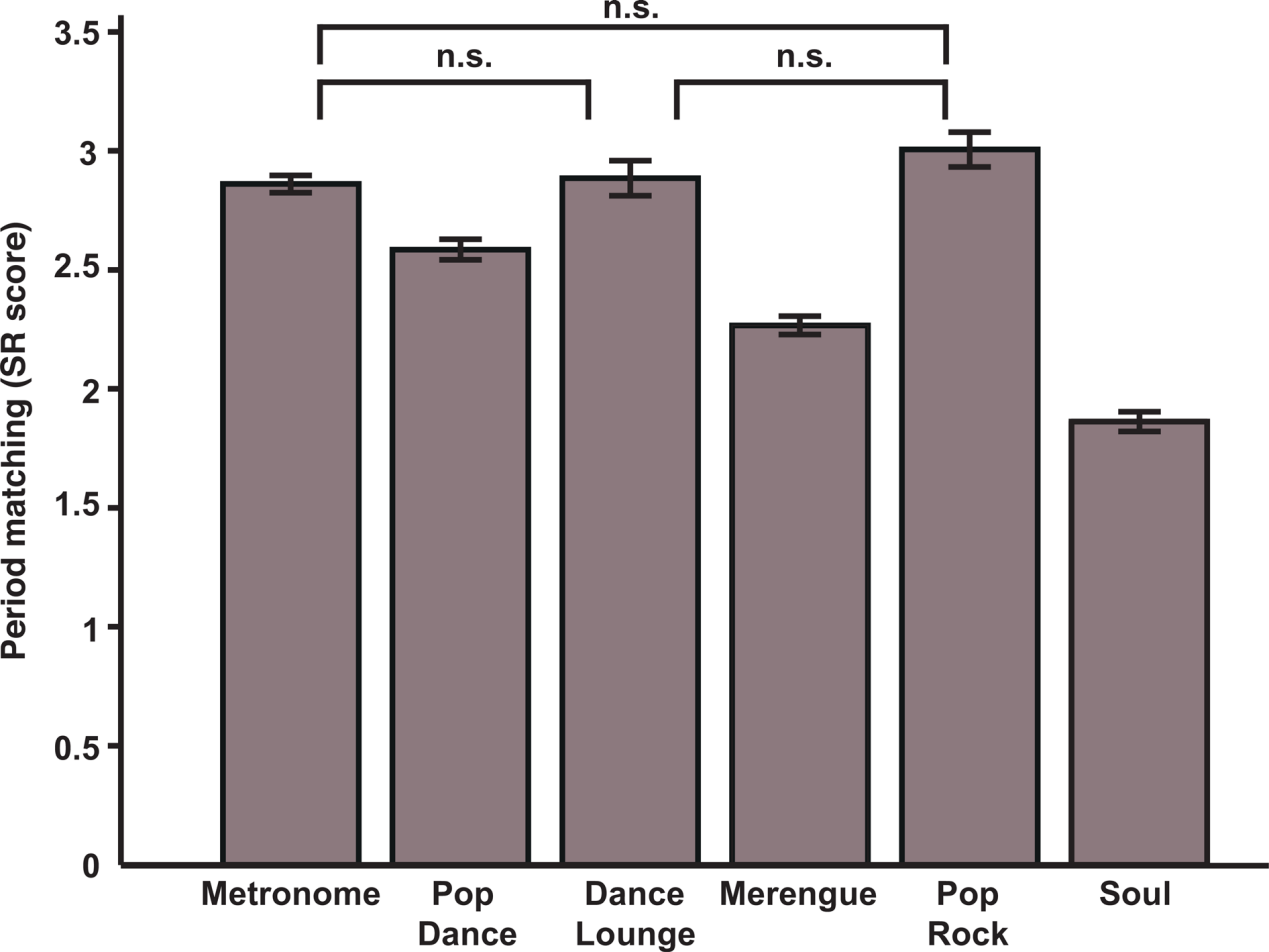
Period matching in Normal Synchronizers' group. Bouncing and clapping scores were averaged for each stimulus and each participant (no interaction between Movement Type and Beat Saliency factors). Error bars represent standard deviations corrected for repeated measures.

Scores for the two Merengue and the two Metronome stimuli were averaged across tempi (no significant difference between tempi). We assessed the impact of movement type (bouncing vs. clapping) and beat saliency (Table 2) on SR by conducting a two-level hierarchical linear regression with Movement Type and Beat Saliency as predictors, and with all data nested within participant. We performed this statistical analysis with the lme4 package [48] in R (http://cran.r-project.org/). A trial synchronized at the beat level contains twice as many responses as a trial synchronized at the two-beat level; this was taken into account by weighting the variance based on the number of responses in each trial, using the *weights* parameter of the *lmer* function. We tested a series of increasingly elaborated models (as recommended by [49]), in which a random intercept was modeled for each participant, and both fixed and random slopes were tested for the main effects of Movement Type and Stimulus Type, as well as their interaction. The final, best-fitting model included a random intercept for participants, fixed effects of both Movement Type and Beat Saliency, and a random slope for Movement Type. All analysis steps leading to the final model and its specifications are provided in S2 Table. According to the fixed effects, SR was predicted significantly by the main effect of Movement Type (*b* = 0.44, *SE* = 0.06, *p* < .001), with clapping being more regular than bouncing, and by the main positive effect of Beat Saliency (*b* = 0.23, *SE* = 0.08, *p* = 0.0087). There was no significant interaction between these two factors (*b* = 0.02, *SE* = 0.05, *p* = 0.68). The significant random slope for Movement Type indicated that the relation between bouncing and clapping scores is different from person to person. When collapsing bouncing and clapping SR, post-hoc comparisons (Bonferroni adjusted, *p* = .05) indicated that all stimuli differed from one another, except for the Metronome, Dance Lounge and Pop Rock. SR to these three stimuli were statistically equivalent, and furthermore were better than for the other musical stimuli. Thus, though beat saliency was a significant positive predictor of SR, some stimuli elicited higher SR than others with relatively higher beat saliency (Fig 2). Interestingly, the two most familiar stimuli (Merengue and Soul) elicited the worse synchronization scores. Note that because stimulus presentation order was kept constant between participants and conditions, there is a possibility that uncontrolled parameters played a role in these findings. Finally, there was a correlation between bouncing and clapping synchronization for music (using the averaged score for the 6 musical stimuli), *r*(82) = 0.39, *p* < 0.001, but not for the metronome (averaged score for the 2 metronome stimuli), *r*(82) = -0.078, *p* = 0.48.

### Poor synchronizers

**Fig 3:**
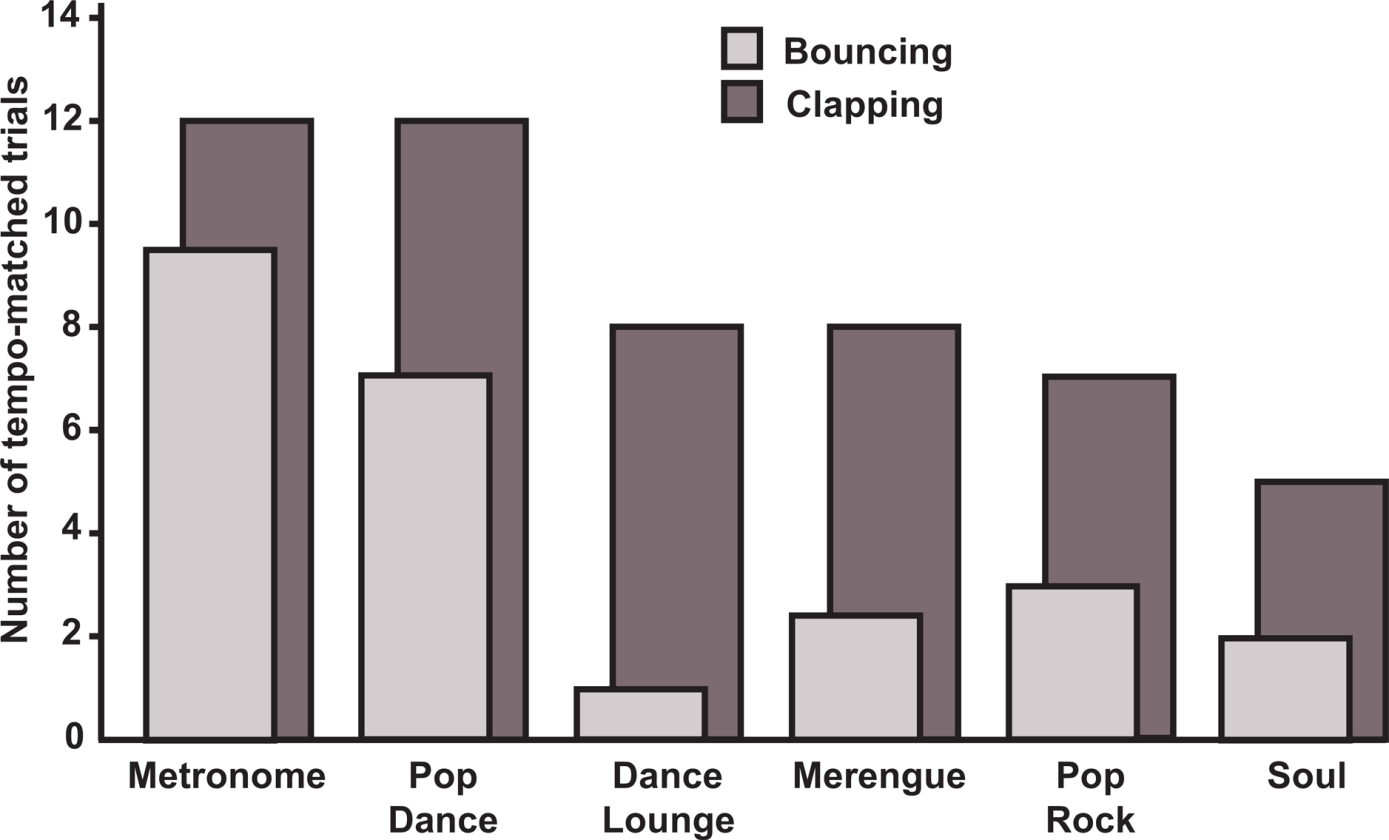
Period matching in Poor Synchronizers' group. We presented the number of success trials (i.e. period-matched), for each movement type and for all stimuli (maximum is 14, i.e. the number of poor synchronizers). The two Merengue and two Metronome were averaged.

The goal of this section is to assess whether the 14 Poor Synchronizers’ profile paralleled the Normal Synchronizers’ profile in terms of the effects for Movement Type and Beat Saliency. Whereas normal synchronization performance could be assessed with synchronization regularity scores, this could not be done in Poor Synchronizers because they had many trial segments with failed synchronization trials (i.e., non-significant Rayleigh tests), and to compare SRs that have been calculated from failed synchronization segments is meaningless because it involves comparing the degree to which performance was random. Therefore, we used proportion of synchronization successes (i.e., trials for which every segment had a significant Rayleigh’s test) as a measure of performance in each movement type and beat saliency condition. This analysis revealed that clapping (*Mdn* = 8.5) was less impaired than bouncing (*Mdn* = 3), *U* = 154.50, *p* = 0.0073. In particular, four Poor Synchronizers (25, 38, 46 and 62) had normal synchronization (i.e., no synchronization failures) when clapping. Like for the Normal Synchronizers' group, beat saliency seemed to play a role: we found a higher proportion of normal performance trials on Metronome and Pop Dance stimuli (i.e., the stimulus with the highest level of beat saliency; Fig 3). At the group level, the Poor Synchronizers’ profile thus seems to parallel that of Normal Synchronizers’. Note that because stimulus presentation order was kept constant between participants and conditions, there is a possibility that uncontrolled parameters played a role in these findings.

At the individual level, Poor Synchronizers presented a variety of behavioural profiles, ranging from synchronization failures on all trials to synchronization failures when bouncing to music only. In order to classify poor synchronizers into subgroups of converging profiles, a cluster analysis was conducted on the proportion of failed synchronization trials for each participant when bouncing to music, bouncing to metronome, clapping to music and clapping to metronome. An agglomerative hierarchical cluster analysis determined that a 4-cluster solution was ideal and cluster centroids were estimated with a *k*-means cluster analysis (MacQueen algorithm). The final centroids for each cluster and Poor Synchronizers’ classification into clusters are presented in Table 4. The first cluster included two Poor Synchronizers, who exhibited poor tempo matching in all of the conditions. The second cluster included four Poor Synchronizers, who presented poor tempo matching when bouncing and clapping to music, but normal performances with the metronome. The impairment thus seemed to emerge for music only. The third cluster included four Poor Synchronizers who were less impaired on clapping as compared to bouncing and less impaired on metronome as compared to music. The fourth and last cluster included four Poor Synchronizers who showed normal clapping performance across all stimuli but impaired performance in bouncing (to both music and metronome). Thus, the impairment in these cases seems to be specific to bouncing.

**Table 4:** Poor Synchronizers' profiles.

Cluster	Participants	Bouncing to Music^1^	Clapping to Music^1^	Bouncing to Metronome^1^	Clapping to Metronome^1^
1	18, 93	0	0	0	0
2	31, 37, 80, 92	16.7	37.7	100	100
3	5, 25, 62, 97	33.5	87.5	100	100
4	6, 38, 46, 53	12.5	83.3	33.5	100

^**1**^Centroïds calculated on the proportion of success trials

In summary, scrutiny of Poor Synchronizer’s data reveals different individual profiles, suggesting that poor synchronization is not a uniform impairment. However, at the group level bouncing is more difficult than clapping and music is more difficult than metronome, suggesting a parallel with the Normal Synchronizer’s group profile. A summary of each Poor Synchronizer’s performance on the Synchronization tasks, the Metric perception test (see results below) and self-paced motor production (see results below) tasks is provided in Supporting Information (S3 Table).

### Self-paced motor production

**Fig 4:**
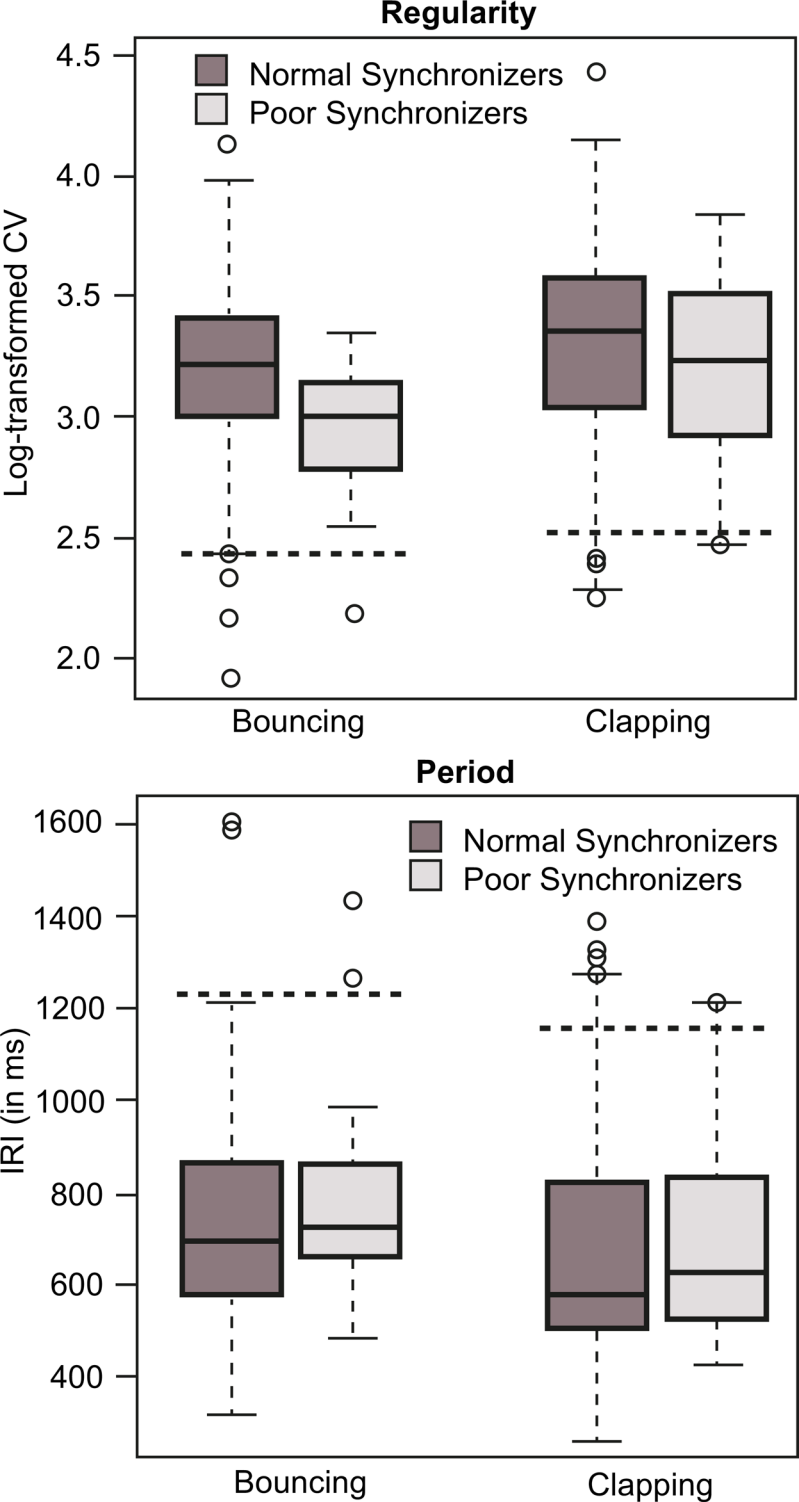
Self-paced motor production. We present produced period (mean inter-bounce/clap-intervals) and regularity (coefficient of variation) in Normal Synchronizers and Poor Synchronizers. For period, dotted horizontal lines show 2 SD above the mean (there were no participants performing below 2 SD below the mean). For regularity, dotted horizontal lines show 2 SD below the mean (i.e. extremely low regularity). Circles indicate outliers (2 SD from the mean).

In order to assess whether Poor Synchronizers were able to maintain regularity in the absence of an external pacing stimulus, we measured their regularity when asked to regularly bounce and clap in silence. The produced rate can be considered to reflect participant’s subjective tempo, called the referent period [50]. We compared Poor Synchronizers’ produced period and regularity to those of Normal Synchronizers to detect potential anomalies of their referent period, such as an extremely slow or an extremely fast tempo, or irregular IRIs. There was one missing file in each of the bouncing and clapping conditions due to equipment error, both for Normal Synchronizers. The mean IRI of the first 30 events was calculated to determine the produced tempo while the coefficient of variation (CV), i.e. standard deviation (SD) of the IRIs divided by the mean IRI, of the first 30 events was used to assess regularity. Normal and Poor Synchronizer group IRIs and CVs are summarized in Fig 4.

#### Normal synchronizers

A paired-sample t-test revealed that the mean IRI was shorter in the clapping condition (*M* = 681, *SD* = 243) compared to the bouncing condition (*M* = 758, *SD* = 237), *t*(79) = 2.6, *p* = 0.011, paralleling the tendency of all participants to bounce at the two-beat level and to clap at the beat-level in the synchronization tasks. Two Normal Synchronizers bounced at a tempo slower than 2 SD from the mean, and four Normal Synchronizers clapped at a tempo slower than 2 SD from the mean.

The distributions of the CV for the bouncing and clapping conditions were consistent with a lognormal function. This was expected because the CV was bounded by zero. To normalize the distribution, we calculated CV's inverse logarithm. This normalized CV was used as the index of regularity (higher values represent lower variability).

Clapping was marginally more regular than bouncing, *t*(79) = 1.96, *p* = 0.054. However, three Normal Synchronizers produced regularity scores more than two SD below the mean in the clapping condition and four Normal Synchronizers did so in the bouncing condition. Self-paced regularity did not predict synchronization regularity, justifying its non-inclusion in the multilevel modeling of the normal synchronization data presented previously. There was no significant correlation between self-paced regularity and synchronization to a metronome (scores averaged across the two tempi), and no significant correlation between self-paced regularity and regularity in synchronization to music (scores averaged across all musical stimuli), all *p* > 0.34. Moderate correlations were obtained when standard deviations of the IRIs rather than CV were considered, but here we report CV because it is the standard measure used in this field (e.g. [51]). No correlations were found between regularity and musical or dance training, all *p* > 0.28.

#### Poor synchronizers

We compared Poor Synchronizers’ self-paced performance to cut-off scores established by the performance of the Normal Synchronizers (mean minus 2 SD).

In the bouncing condition, self-paced tempo did not differ between Poor Synchronizers and Normal Synchronizers, *t*(92) = 0.65, *p* = 0.51. Two Poor Synchronizers (18 and 53) were slower than the cut-off. In the clapping condition, self-paced tempo did not differ between Poor Synchronizers and Normal Synchronizers, *t*(92) = 0.4, *p* = 0.69. One Poor Synchronizer (80) was slower than the cut-off.

In the bouncing condition, regularity was significantly worse in Poor Synchronizers than in Normal Synchronizers, *t*(92) = 2.6, *p* = 0.011. One Poor Synchronizer (80) had regularity below the cut-off for bouncing. However, in the clapping condition, this participant’s score was above the mean of the normal synchronization group, indicating that participant 80’s poor regularity was limited to bouncing. In the clapping condition, regularity was not significantly different between Poor Synchronizers and Normals, *t*(92) = 0.86 *p* = 0.39. One Poor Synchronizer (31) presented regularity below the cut-off.

### Metric perception

The Normal Synchronizers’ mean score was 27.4 out of 30 (*SD* = 3.65). This distribution is highly asymmetric with negative skewness and a mode of 30. Therefore, as a group, Normal Synchronizers performed well on this task. However, six normal synchronizers performed poorly with a score inferior to two standard deviations below the mean (cut-off: 20). Note that there is not enough variability in the normal group to perform a correlation with synchronization scores. Moreover, no correlation was found between synchronization and the off-beat detection test of the on-line test of amusia.

The mean score of the 14 poor synchronizers was 24.07 (*SD* = 5.41) and was significantly worse than normal performance, *t*(94) = 2.91, *p* = 0.0045. Two Poor Synchronizers, 6 and 46, obtained a score inferior to the cut-off of 20, with scores of 13 and 12, respectively. The other Poor Synchronizers obtained a score superior to the cut-off, and in particular participants 25, 62 and 93 scored above the Normal Synchronizers’ mean.

## Discussion

In the present study, we examined the role of movements and beat saliency in synchronization to music using naturalistic stimuli and movements. We found that the vast majority of young adults are able to match their bounces and claps to the tempo of musical and metronome stimuli, in line with a previous study from our lab [34]. Here, we further show that the matching was less accurate in bouncing than in clapping. Several factors may account for this finding. Bouncing requires more force or physical endurance because each bounce requires the individual to work against gravity to propel the trunk upwards. Indeed, previous work has demonstrated that forearm movement synchronization is less stable when performed against gravitational forces, regardless of the movement’s direction (up or down) [52]. Another possible factor is the absence of auditory and tactile feedback in bouncing compared to clapping, whereby the physical impact between the hands produces a sound for each clap. Sensory feedback plays an important role in the anticipatory timing of synchronized movements [53,54]. During bouncing, both gravity and the absence of sensory feedback may have counteracted the contribution of vestibular stimulation, which is known to play a role in meter perception [17]. Synchronization may be better facilitated by sensory feedback, as is present in clapping, than it is by vestibular stimulation, as is present in bouncing.

Clapping, however, does not only benefit from sensory feedback; it is also a discrete movement. Previous research has shown that motor control develops earlier for discrete than continuous forms of movement during childhood [55] and that the variability of movement timing during synchronization is lower for discrete than continuous forms of movement [56,57]. Therefore, the fact that clapping is a more discrete movement than bouncing may explain why synchronization was more accurate in clapping. Further research is required to disentangle the respective role of discreteness and feedback in synchronization.

In addition to the difference in the accuracy of tempo matching between bouncing and clapping, the two forms of movements often occurred at different levels. Specifically, there was a tendency to clap to every beat but to bounce to every other beat. The latter result is consistent with the observation that the torso moves at higher metrical level (slower tempo) than the arms in dancing [58]. The authors of the study interpreted this in terms of the body’s inertial and biomechanical properties, with the torso having a higher period of oscillation than the extremities. In our case, one alternative and novel explanation may be that bouncing to every other beat is a way to “discretize” the time course of the bouncing motion. Whereas bouncing on every beat requires continuous movement, bouncing half as fast allows for discrete movement by introducing a short break between each bounce. Since separate mechanisms seem to drive discrete and continuous forms of SMS, and better synchronization accuracy is associated with the former, the discretization observed with bouncing may reflect a natural tendency to use discrete movements as a strategy for optimal synchronization.

Bouncing and clapping were found to be similarly affected by the musical beat saliency. This result suggests that higher beat saliency facilitates perceptual beat extraction, thereby removing one source of cognitive load on the SMS process. However, synchronization performance did not strictly follow the beat saliency gradient. For instance, tempo-matching was more accurate to the Dance Lounge and Pop Rock stimuli than to the metronome, which has the most salient beat. Other factors such as groove may play a role. As mentioned in the introduction, high-groove music elicits better SMS coupling than low-groove music [20,25] and groove is associated with pleasure, which is an important component of musical engagement [59,60]. It is thus possible that higher beat saliency was associated with better performances not only because beat extraction was easier but also because it was much more enjoyable. Groove might in particular explain why the metronome, which was given the highest ratings of beat saliency, did not lead to the highest synchronization scores. Not only a metronome is unlikely to elicit much pleasure, but previous work indicates that inter-beat event density (of which the metronome has none) is highly correlated with groove [39].

In sum, optimizing the way researchers measure SMS in participants depends on the specific research question under study. Clapping seems optimal for the characterization of the fine-tuning of SMS. In contrast, bouncing constitutes a more challenging task and may therefore be more sensitive to individual differences. Indeed, all 14 individuals (out of 100) who exhibited poor synchronization performance did so in bouncing, but not necessarily in clapping. Qualitatively, poor synchronization paralleled normal performance, with clapping being better than bouncing and better for music with high beat saliency. These results support the earlier suggestion [61] that poor synchronization may correspond to the low tail of a normal distribution of beat perception abilities. It is also consistent with Poor Synchronizers' lower performance, as compared to Normal Synchronisers, on the MBEA metric test, and the recent observation that poor beat perceivers have weak period-matching abilities compared to good beat perceivers [25].

The present findings question the methods currently used to identify individuals with synchronization difficulties. So far the existing test batteries assess synchronization abilities through finger tapping (e.g., [37,62,63]). Our findings suggest that researchers may be missing whole categories of individuals showing synchronization difficulties, as would have been the case in the present study if we had used clapping only. Therefore, the development and validation of test batteries assessing synchronization via multiple movement forms should be an important goal for researchers in this field. In particular, the recent spread of portable devices containing accelerometers (e.g. smartphones) in the general population may constitute an avenue for the study of synchronization impairments through multiple types of movements on a very large scale.

To conclude, our results show that not all movement types and musical stimuli are equivalent when it comes to synchronizing body motion to external rhythms. A goal for future studies will be to understand why synchronization is more accurate via certain movement types over others, and whether these effects reflect distinct underlying mechanisms or rather a continuum of difficulty along a single mechanism. Indeed, the heterogeneity observed in our poor synchronizers’ sample calls for clarification of the origin of these difficulties, as well as their specificity to music (as compared to speech and other non-auditory modalities).

## Supporting Information

S1 Fig Wii set-up. Clapping (left) and bouncing (right)
(PDF)

S1 Table Description of Normal synchronizers' performances. Log-transformed circular variance mean score (standard deviation) for all conditions (Metronome and Merengue averaged). The higher the score the better the performance. The log-transformed coefficient of variation mean score is also provided.
(DOCX)

S2 Table Model specifications for bouncing and clapping synchronization. Prediction of SR by Movement Type and Beat Saliency factors in Normal Synchronizers.
(DOCX)

S3 Table Description of Poor Synchronizers’ performances. A ‘-‘ indicates a failure to match the tempo of at least three (out of six) of the musical trials, or a failure to match the tempo of both metronome trials. A ‘+ /-‘ indicates a failure to match the tempo of one or two of the musical trials or one of the metronome trials. For Spontaneous motor production, only singular performances are described. For the Metric Perception task, scores below the cut-off are in bold.
(DOCX)
