# Supplementary figures and images for: Keeping the Beat: A Large Sample Study of Bouncing and Clapping to Music
Source: PLoS One. 2016 Jul 29;11(7):e0160178. doi: 10.1371/journal.pone.0160178 (PMC4966945; doi:10.1371/journal.pone.0160178)

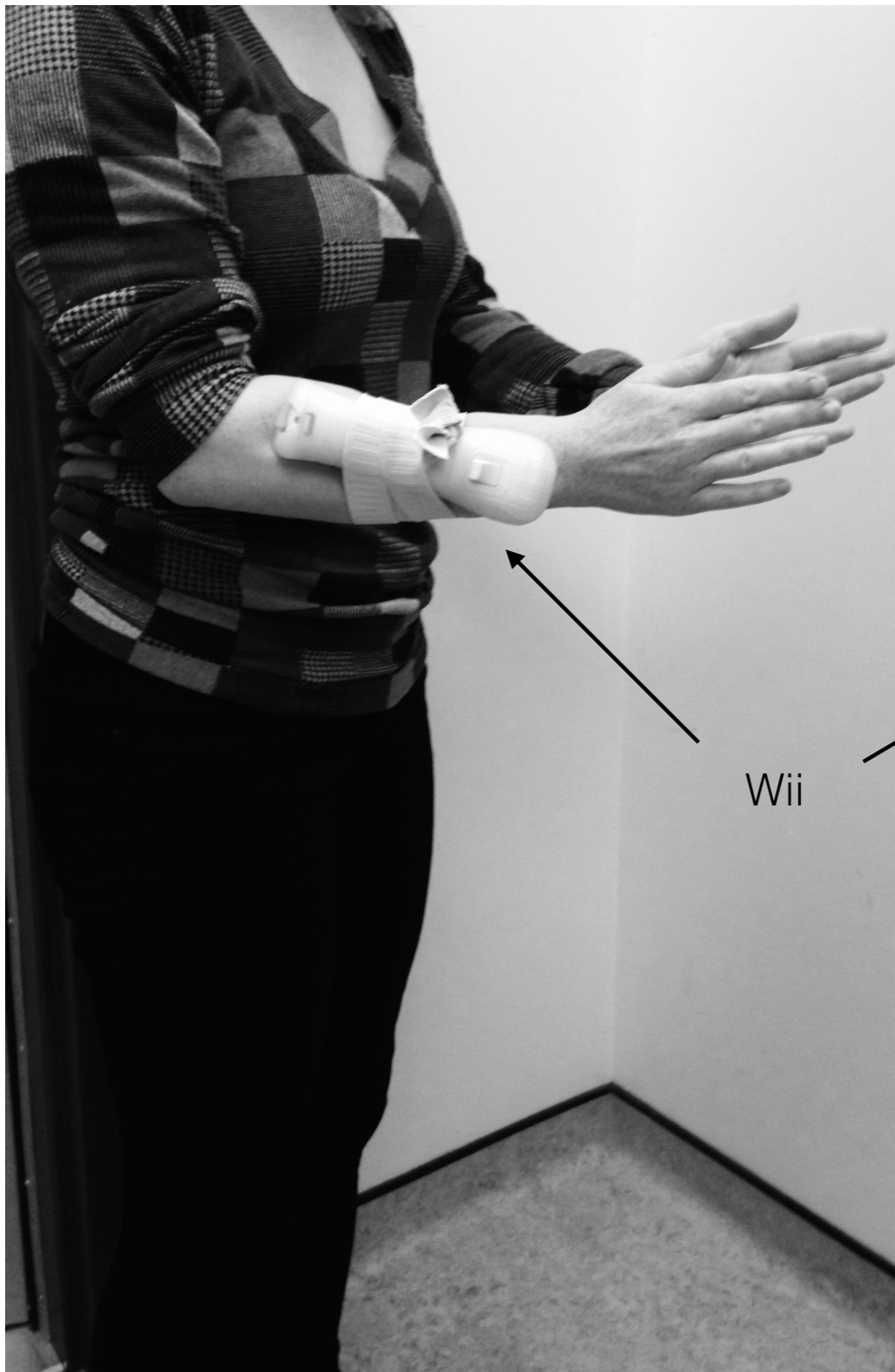

Wii

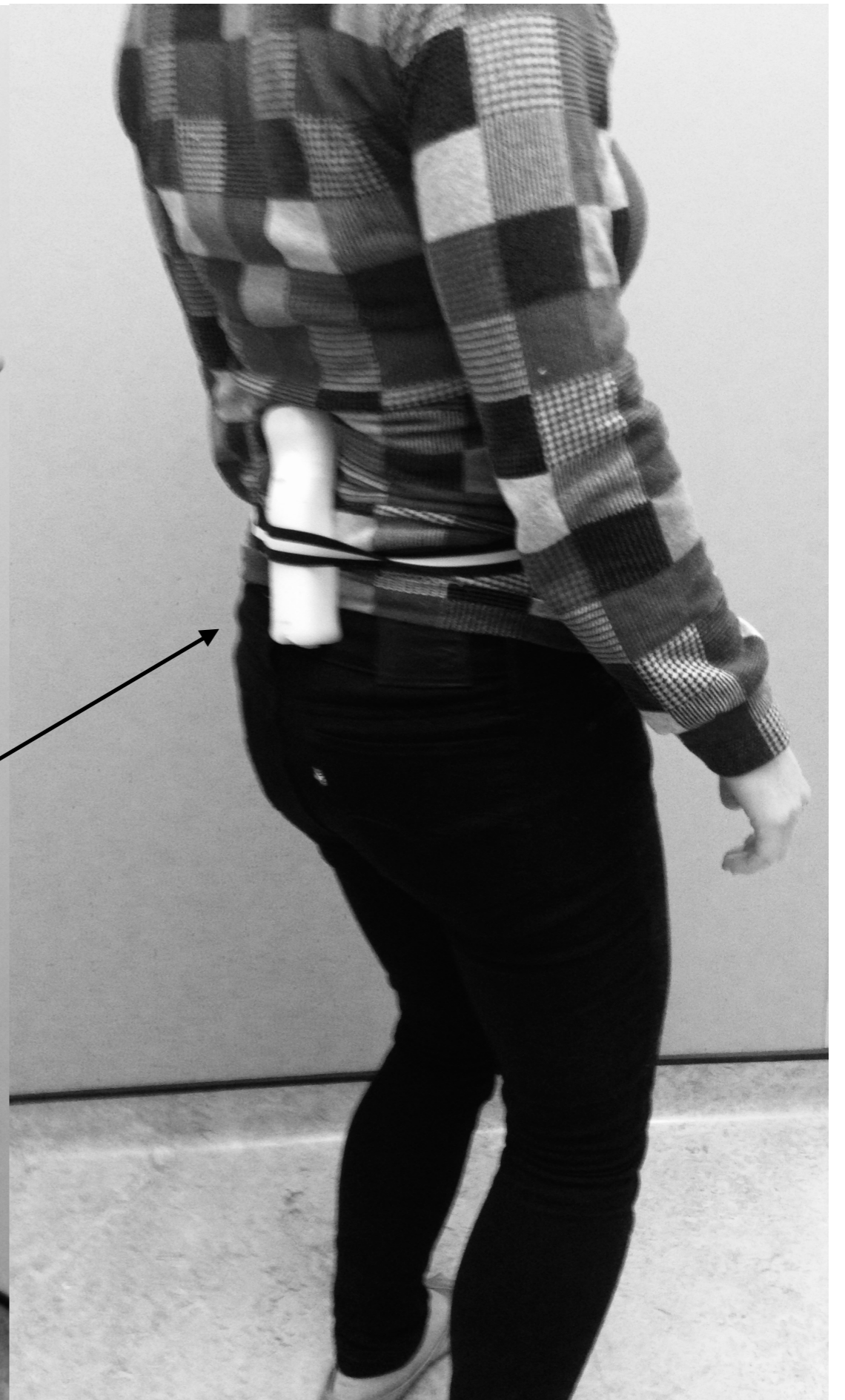

Supplement: S1 Fig — Clapping (left) and bouncing (right) (PDF) [file pone.0160178.s001.pdf]
