# Supplementary material for: Keeping the Beat: A Large Sample Study of Bouncing and Clapping to Music
Source: PLoS One. 2016 Jul 29;11(7):e0160178. doi: 10.1371/journal.pone.0160178 (PMC4966945; doi:10.1371/journal.pone.0160178)
Supplement: S1 Table — Log-transformed circular variance mean score (standard deviation) for all conditions (Metronome and Merengue averaged). The higher the score the better the performance. The log-transformed coefficient of variation mean score is also provided. (DOCX) [file pone.0160178.s002.docx]

**S1 Table. Description of Normal synchronizers' performances.**

|  |  | **Metro-nome** | **Pop Dance** | **Dance Lounge** | **Meren-gue** | **Pop**  **Rock** | **Soul** |
| --- | --- | --- | --- | --- | --- | --- | --- |
| **Bouncing** | Coefficient  of Variation  Circular Variance | 2.93 (0.40)  2.54 (0.60) | 3.00 (0.40)  2.44 (0.63) | 2.97 (0.43)  2.56 (0.77) | 2.99 (0.35)  2.12 (0.63) | 2.88 (0.48)  2.59 (0.91) | 2.90 (0.50)  1.80 (0.65) |
| **Clapping** | Coefficient  of Variation  Circular Variance | 3.16 (0.39)  3.07 (0.44) | 3.17 (0.38)  2.73 (0.47) | 2.99 (0.54)  3.21 (0.63) | 3.12 (0.43)  2.28 (0.50) | 3.08 (0.56)  3.42 (0.89) | 3.22 (0.49)  1.93 (0.66) |
