# Supplementary material for: Keeping the Beat: A Large Sample Study of Bouncing and Clapping to Music
Source: PLoS One. 2016 Jul 29;11(7):e0160178. doi: 10.1371/journal.pone.0160178 (PMC4966945; doi:10.1371/journal.pone.0160178)
Supplement: S2 Table — Prediction of SR by Movement Type and Beat Saliency factors in Normal Synchronizers. (DOCX) [file pone.0160178.s003.docx]

**S2 Table. Model specifications for bouncing and clapping synchronization.**

| Model | ***b* (*SE*)**  **(fixed effects)** | | | | | | | | | Model Statistics  **χ^2^(*df* = 1)** |
| --- | --- | --- | --- | --- | --- | --- | --- | --- | --- | --- |
| Step 0 :  Data ~ (1\| participant) | 2.61(0.04)*** | | | | | | | | |  |
| Step 1 :  Data ~ Beat Saliency + (1\| participant) | Intercept | | | | Beat Saliency | | | | | 65.48*** |
|  | 2.62(0.04)*** | | | | 0.25(0.03)*** | | | | |  |
| Step 2 :  Data ~ Beat Saliency + Mov. Type + (1\| participant) | Intercept | | | Beat Saliency | | | Mov. Type | | | 84.98*** |
|  | 1.99(0.08)*** | | | 0.26(0.03)*** | | | 0.43(0.03)*** | | |  |
| Step 3 :  Data ~ Beat Saliency * Mov. Type + (1\| participant) | Intercept | | Beat Saliency | | Mov. Type | | | Beat Saliency*Mov. Type | | 0.09 |
|  | 1.99(0.08)*** | | 0.23(0.09)** | | 0.43(0.05)*** | | | 0.02(0.06) | |  |
| Step 4 :  Data ~ Beat Saliency * Mov. Type + (1+ Mov. Type \| participant) | Intercept | Beat Saliency | | | | Mov. Type | | | Beat Saliency*Mov. Type | 29.83*** |
|  | 1.97(0.11)*** | 0.23(0.08)** | | | | 0.44(0.06)*** | | | 0.02(0.05) |  |

**Note:** ****p* < .001, ***p* < .01, **p* < .05, †*p* < .10;
