# Supplementary material for: Keeping the Beat: A Large Sample Study of Bouncing and Clapping to Music
Source: PLoS One. 2016 Jul 29;11(7):e0160178. doi: 10.1371/journal.pone.0160178 (PMC4966945; doi:10.1371/journal.pone.0160178)
Supplement: S3 Table — A ‘-‘ indicates a failure to match the tempo of at least three (out of six) of the musical trials, or a failure to match the tempo of both metronome trials. A ‘+ /-‘ indicates a failure to match the tempo of one or two of the musical trials or one of the metronome trials. For Spontaneous motor production, only singular performances are described. For the Metric Perception task, scores below the cut-off are in bold. (DOCX) [file pone.0160178.s004.docx]

**S3 Table. Description of Poor Synchronizers’ performances.**

| Participant | Synchronization | | | | Spontaneous Motor Production | MBEA Meter Test  (cut-off=22) |
| --- | --- | --- | --- | --- | --- | --- |
|  | Bouncing | | Clapping | |  |  |
|  | Musique | Metro-nome | Musique | Metro-nome |  |  |
| P18 | - | - | - | - | Slow (bouncing) | 24 |
| P93 | - | - | - | - |  | **20** |
| P31 | - | + | - | + | Regularity - (clapping) | 27 |
| P37 | - | + | - | + |  | 26 |
| P80 | - | + | - | + | Regularity – (bouncing) & slow (clapping) | 25 |
| P92 | - | + | - | + |  | 30 |
| P05 | - | + | + /- | + |  | 26 |
| P25 | - | + | + /- | + |  | 30 |
| P62  P97 | -  - | +  + | +  + /- | +  + |  | 28  26 |
| P06  P38 | -  - | + /- | + /- | +  + |  | **13**  27 |
| P46  P53 | -  - | + /-  - | +  +/- | +  + | Slow | **12**  22 |
